# A haplotype‐resolved reference genome of *Quercus alba* sheds light on the evolutionary history of oaks

**DOI:** 10.1111/nph.20463

**Published:** 2025-02-11

**Authors:** Drew A. Larson, Margaret E. Staton, Beant Kapoor, Nurul Islam‐Faridi, Tetyana Zhebentyayeva, Shenghua Fan, Jozsef Stork, Austin Thomas, Alaa S. Ahmed, Elizabeth C. Stanton, Allan Houston, Scott E. Schlarbaum, Matthew W. Hahn, John E. Carlson, Albert G. Abbott, Seth DeBolt, C. Dana Nelson

**Affiliations:** ^1^ Department of Biology Indiana University Bloomington IN 47405 USA; ^2^ Department of Entomology and Plant Pathology University of Tennessee Knoxville TN 37996 USA; ^3^ USDA Forest Service, Southern Research Station College Station TX 77843 USA; ^4^ Department of Ecology and Conservation Biology Texas A&M University College Station TX 77843 USA; ^5^ Department of Forestry and Natural Resources University of Kentucky Lexington KY 40546 USA; ^6^ Department of Horticulture University of Kentucky Lexington KY 40546 USA; ^7^ Oak Ridge Institute for Science and Education (ORISE) USDA Forest Service, Southern Research Station Lexington KY 40546 USA; ^8^ Genome Science and Technology University of Tennessee Knoxville TN 37996 USA; ^9^ School of Natural Resources University of Tennessee Knoxville TN 37996 USA; ^10^ Department of Computer Science Indiana University Bloomington IN 47405 USA; ^11^ Department of Ecosystem Science and Management Pennsylvania State University University Park PA 16802 USA; ^12^ Abbott Tree Farm and Research Consultants Cape Vincent NY 13618 USA; ^13^ James B. Beam Institute for Kentucky Spirits University of Kentucky Lexington KY 40546 USA; ^14^ USDA Forest Service, Southern Research Station Lexington KY 40546 USA

**Keywords:** comparative genomics, gene family evolution, genome assembly, phylogenetic tree, population structure, *Quercus alba* (white oak)

## Abstract

- White oak (*Quercus alba*) is an abundant forest tree species across eastern North America that is ecologically, culturally, and economically important.
- We report the first haplotype‐resolved chromosome‐scale genome assembly of *Q. alba* and conduct comparative analyses of genome structure and gene content against other published Fagaceae genomes. We investigate the genetic diversity of this widespread species and the phylogenetic relationships among oaks using whole genome data.
- Despite strongly conserved chromosome synteny and genome size across *Quercus*, certain gene families have undergone rapid changes in size, including defense genes. Unbiased annotation of resistance (R) genes across oaks revealed that the overall number of R genes is similar across species – as are the chromosomal locations of R gene clusters – but, gene number within clusters is more labile. We found that *Q. alba* has high genetic diversity, much of which predates its divergence from other oaks and likely impacts divergence time estimations. Our phylogenetic results highlight widespread phylogenetic discordance across the genus.
- The white oak genome represents a major new resource for studying genome diversity and evolution in *Quercus*. Additionally, we show that unbiased gene annotation is key to accurately assessing R gene evolution in *Quercus*.

## Introduction

Oaks (*Quercus* spp.) are important members of ecosystems throughout much of the world (Kremer & Hipp, 2020). In eastern North America, white oak (*Quercus alba*) is a keystone species and is one of the most abundant forest trees across much of its range (Rogers, 1990; Fralish, 2004). In addition to its ecological and cultural importance (Abrams, 2003; Bocsi *et al*., 2021a,b; Stringer & Morris, 2022), white oak has significant economic importance, including a number of high‐value timber applications and as the primary species used to cooper barrels for aging distilled spirits (Stringer & Morris, 2022; Dhungel *et al*., 2023). However, few studies have addressed the genomic diversity of *Q. alba*, and a lack of available genetic and genomic resources currently presents barriers to furthering the understanding of white oak biology and evolutionary history.

*Quercus* (Fagaceae) comprises *c*. 500 species, often divided into two subgenera: *Cerris* and *Quercus* (Hipp *et al*., 2020). The latter is typically further divided into the white oaks (section *Quercus*), to which *Q. alba* belongs, and the red oaks (section *Lobatae*). The phylogeny of oaks has been the focus of several recent studies utilizing reduced representation genome sequencing (Sork *et al*., 2016; Hipp *et al*., 2020; Manos & Hipp, 2021), which have clarified some relationships within section *Quercus*. However, phylogenetic inference in oaks is likely complicated by the suggested prevalence of hybridization and introgression in the group (e.g. McVay *et al*., 2017; Lazic *et al*., 2021).

The first published oak genome was that of *Quercus robur* L. (Plomion *et al*., 2016a), the pedunculate oak, which is common throughout western Eurasia. To date, there have been at least 11 *Quercus* species with published chromosome‐scale genomes, including four annotated genomes from the white oak clade (Plomion *et al*., 2016a; Ai *et al*., 2022; Han *et al*., 2022; Liu *et al*., 2022, 2024; Sork *et al*., 2022; Zhou *et al*., 2022; Kapoor *et al*., 2023; L. Wang *et al*., 2023; W. Wang *et al*., 2023). This growing number of annotated genomes allows for comparative analyses of gene content and inferences of genome evolution across the oak phylogeny.

Disease resistance‐related genes (R genes) have been a focus of genomic studies on oaks and other tree species because of their central role in plant immunity to pathogens (Plomion *et al*., 2018; Ai *et al*., 2022; Sork *et al*., 2022). Resistance genes confer defense against various viral, bacterial, and eukaryotic pathogens by encoding proteins that recognize pathogen‐related molecules and trigger downstream immune responses. Plomion *et al*. (2018) suggested that an expansion of R genes might be at least partly responsible for allowing tree species to live for multiple centuries. Ai *et al*. (2022) found that the *Quercus mongolica* Fisch. ex Ledeb. genome assembly contained far fewer putative R genes than the earlier genome assemblies of *Quercus lobata* Née, *Q. robur* L., and *Quercus suber* L. However, the history of how these R gene families have evolved across the oak phylogeny remains poorly understood.

In addition to providing insights into fundamental questions about plant evolution, improving the understanding of genomes across the white oak clade may benefit tree breeding and genetic improvement efforts and help land managers plan for and address global change. *Q. alba* faces declining seedling recruitment in many parts of its range (Dhungel *et al*., 2023), which may have implications for ecosystem function throughout eastern North America. Furthermore, anthropogenic climate change is causing a mismatch between populations and their historical climates (Piao *et al*., 2019; Kijowska‐Oberc *et al*., 2020). The amount of standing genetic variation and the extent to which populations are locally adapted will have implications for the response of *Q. alba* and other white oak species to global climate change.

Here, we report the first assembled genome of *Q. alba* and use this new resource to study the evolution of oak genomes. Specifically, we address the following questions. What is the extent of genetic diversity and population differentiation within *Q. alba*? Are previous phylogenetic hypotheses for the relationships among oak species supported by whole genome data? How have gene content and disease resistance genes evolved during the history of *Quercus* and related taxa? The answers to these questions will provide a roadmap for future work on the white oak group as a model clade for studying genome evolution and adaptation in highly outcrossing forest trees.

## Materials and Methods

### Reference genome

**Fig. 1:**
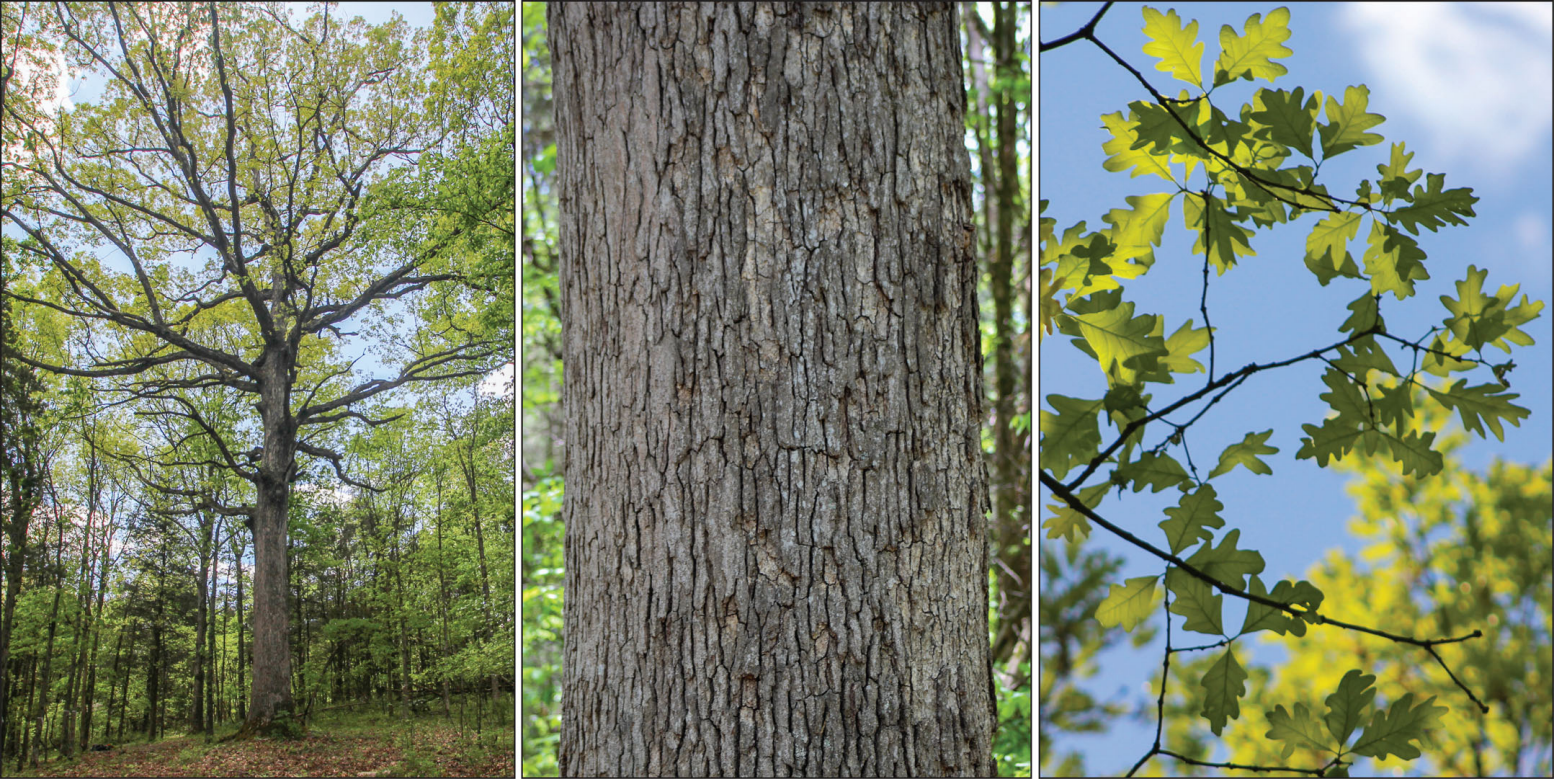
MM1, the *Quercus alba* individual sequenced for the genome assembly, growing at Star Hill Farm, Loretto, KY, USA. Photograph attribution: D. Larson.

An individual of *Q. alba* L. in a forest stand near Loretto, Kentucky, USA (37°39.0583′, −85°21.2615′), referred to as MM1 (Fig. 1), was sampled with the permission of the landowners. Following high‐molecular‐weight DNA extraction and sequencing, PacBio Sequel II HiFi and Hi‐C (Phase Genomics, Seattle, WA, USA) data were assembled with Hifiasm v.0.16.1 (Cheng *et al*., 2021) and scaffolded with 3D‐DNA v.201008 (Dudchenko *et al*., 2017; Supporting Information Methods S1). Two resolved haplotypes were produced, herein referred to as hapA and hapB. The plastome and mitochondrial genomes of MM1 were assembled and annotated separately (Methods S1). To assess genome quality, Busco v.5.2.2 and the embryophyta_odb10 database were used to analyze the hapA and hapB assemblies (Manni *et al*., 2021). The synteny and structural variations of the haplotypes were analyzed with the Synteny and Rearrangement Identifier (SyRI) v.1.5.4 (Goel *et al*., 2019) based on a mapping of hapB to hapA with Minimap2 v.2.24 (Li, 2018) and visualized with plotsr v.0.5.1 (Goel & Schneeberger, 2022). Braker2 and Tsebra were used to independently annotate the hapA and hapB assemblies (Bruna *et al*., 2021; Gabriel *et al*., 2021; Methods S1). RNA sequencing was performed on several tissue types from four individuals and analyzed alongside RNA data obtained from NCBI (SRR006309–SRR006312) (Methods S1). A genetic linkage map was constructed based on 184 full‐ and half‐sib *Q. alba* individuals (Methods S1).

The locations of rRNA arrays in hapA and hapB were identified with RNAmmer (Lagesen *et al*., 2007). Fluorescence *in situ* hybridization (FISH) with rRNA oligonucleotide probes was conducted following the methods of Kapoor *et al*. (2023). R gene domains and categories of the annotated genes were determined using interproscan and the NLR_classification script from FindPlantNLRs (Chen *et al*., 2023). Next, the full FindPlantNLRs pipeline, which ingests the raw genome without gene or repeat annotation, was used to refine R gene identification. Visualizations of R gene locations were created with RIdeogram (Hao *et al*., 2020), and clusters were formed by aggregating genes within 200 kb of each other. Mechanisms of gene duplication were inferred by the MCScanX duplicate gene classifier tool (Wang *et al*., 2012) of the hapA assembly against itself, resulting in the assignment of five categories of gene expansion type: (1) whole genome/segmental duplication where a set of collinear genes are found duplicated in collinear blocks, (2) tandem duplication where duplicate genes are adjacent to each other, (3) proximal duplication where genes are not adjacent but are in nearby chromosomal regions, (4) dispersed duplications where duplicated genes are found but do not fit the criteria for other categories, and (5) single copy (‘singleton’) genes that have no identified duplication from other genes.

### Population genomics

We sequenced 16 unrelated *Q. alba* individuals (four individuals each of Wisconsin, OH, Indiana, and Mississippi provenances), as well as two additional *Q. alba* individuals from Kentucky (Tables S1, S2; Methods S1). We also included the MM1 tree in our population genetic analyses; the sequence data utilized for this individual were the two runs of PacBio HiFi long reads described previously. Sequencing data were analyzed with Gatk (McKenna *et al*., 2010; DePristo *et al*., 2011; Van Der Auwera *et al*., 2013) following read trimming and processing (Li *et al*., 2009; Li & Durbin, 2009; Andrews, 2010; Jiang *et al*., 2014; Sim *et al*., 2022; Broad Institute, 2023). To conduct genetic clustering with Structure v.2.3.4 (Pritchard *et al*., 2000), 10 000 SNPs were randomly selected after additional processing (Methods S1). We summarized our Structure results with the clumpak online server and default settings (Kopelman *et al*., 2015). We also conducted a principal component analysis (PCA) with Plink v.2.0 (Purcell *et al*., 2007) and visualized the first two principal components, which correspond to the first two eigenvectors, using the ggplot2 library in R (R Core Team, 2013; Wickham, 2016). Pairwise *F*_ST_ values were calculated with the Reich–Patterson estimator (Reich *et al*., 2009) as implemented by Junker *et al*. (2020) in R (Methods S1). Genome‐wide nucleotide diversity (π) was calculated using Vcftools v.0.1.13 (Danecek *et al*., 2011; Methods S1).

### Phylogenomics

A phylogenomic dataset was assembled using data from several sources including new sequencing of seven species of section *Quercus* and NCBI (Tables S1, S2; Methods S1). We called variants using the *mpileup*, *call*, and *consensus* commands in BCFtools (Li, 2011) and the hapA reference to produce whole genome pseudo‐reference sequences for each sample, masked repetitive DNA, and randomly selected one allele at heterozygous sites. As a final step to prepare our pseudoreference sequences, we used the referee package (Thomas & Hahn, 2019) to calculate genotype quality scores for each site and masked sites where the final base call was not supported (Methods S1). To infer phylogenetic relationships, we generated a matrix of whole genome alignments (i.e. all 12 chromosomes, excluding unplaced scaffolds), which included 37 individuals from 19 species of *Quercus* and one individual of *Lithocarpus* Blume as an outgroup. A maximum likelihood phylogenetic tree was estimated with IQ‐Tree v.2.2.0; site concordance factors (sCF) were calculated with the ‘‐‐scf’ option and 1000 quartet replicates (Minh *et al*., 2020a,b; Mo *et al*., 2023). To investigate the shared genetic variation between *Q. alba* and other oak species, we used the same pseudoreference matrix (Methods S1). An ultrametric phylogeny was generated with branch lengths scaled to time using the penalized likelihood approach as implemented in treePL (Sanderson, 2002; Smith & O'Meara, 2012) and a calibration for the crown age of *Quercus* at 56 million years ago (Hofmann, 2010; Hofmann *et al*., 2011; Hipp *et al*., 2020), after accounting for ancestral polymorphism by using our estimate of π in *Q. alba* (Edwards & Beerli, 2000; Methods S1). We also generated phylogenetic trees from nonoverlapping 5 kb windows and estimated a species tree from the 12 091 resulting window trees with Astral v.5.7.7 (Zhang *et al*., 2017) and default settings. Phylogenetic conflict using gene concordance factors (gCF) for both the maximum likelihood and Astral tree was calculated with IQ‐Tree; phylogenetic conflict using bipartitions was calculated for the maximum likelihood tree with PhyParts (Smith *et al*., 2015; Methods S1). We also investigated phylogenetic relationships of the chloroplast and mitochondrial genomes of these samples (Methods S1).

### Comparative genomics

Syntenic structure for eight oak genomes and those of five other Fagaceae species was assessed using SyRI as described above, with hapA as the reference (Methods S1). A syntenic block analysis was conducted with 11 species of Fagales by using OrthoFinder v.2.5.4 (Emms & Kelly, 2019) on primary proteins, followed by syntenic block identification and duplicate gene classification by MCScanX (Wang *et al*., 2012; Methods S1). Gene families were determined in hapA and primary protein sets from seven *Quercus* species by running GeneSpace (Lovell *et al*., 2022), which uses OrthoFinder v.2.5.4 and MCScanX. Expansion and contraction of gene families was determined with Cafe5 (Mendes *et al*., 2020) and the base model. *De novo* annotation of R genes was conducted for seven *Quercus* genomes using the FindPlantNLRs pipeline (Chen *et al*., 2023) and visualized with RIdeogram (Hao *et al*., 2020). A summary of the taxa included in each comparative genomic analysis is shown in Fig. S1.

## Results

### Haplotype‐resolved genome assembly

The reference genome for *Q. alba* L. (tree MM1, Fig. 1) was assembled with PacBio HiFi (circular consensus sequencing) reads with 45× haploid genome coverage and an average read length of 20 753 bases (Table S2). Assembly and scaffolding were further improved by a Hi‐C library, resulting in two resolved haplotypes (i.e. hapA and hapB). HapA includes 763 contigs spanning 794 299 596 bases (L50:29; N50:8.3 Mb), and 300 of these contigs, spanning 97.0% of the total bases, are scaffolded into 12 chromosomes (Table 1). HapB is similarly complete with 563 contigs (L50:29; N50:8.9 Mb) spanning 792 297 883 bases with 215 contigs representing 97.0% of the total bases scaffolded into 12 chromosomes. Busco analysis of the unannotated genomes using 1614 embryophyta conserved genes found that over 98% were present and complete in both haplotypes, and only 5% were complete and duplicated. A complete chloroplast and a draft mitochondrial genome in two scaffolds were also assembled for MM1 (Figs S2, S3).

**Table 1:** Assembly and Busco statistics from hapA and hapB of *Quercus alba*, assessed for the total assembly (All) and the scaffolds placed into the 12 chromosomes (Chrs).

		HapA All	HapA Chrs	HapB All	HapB Chrs
Contigs	Number	763	300	563	215
	Total bases	794 299 596	770 521 868	792 297 883	768 546 887
	L50	29	28	29	28
	N50	8.3 Mb	8.4 Mb	8.9 Mb	8.9 Mb
Scaffolds	Number	417	12	309	12
	Total bases	794 470 984	770 665 549	792 424 023	768 647 478
Protein‐coding genes	Number	42 955	42 489	42 412	42 150
Buscos	Complete (%) Buscos	1592 (98.6%)	1579 (97.8%)	1591 (98.6%)	1587 (98.3%)
	Complete and single‐copy	1517	1505	1526	1522
	Complete and duplicated	75	74	65	65
	Fragmented	9	13	14	14
	Missing	10	22	9	13

**Fig. 2:**
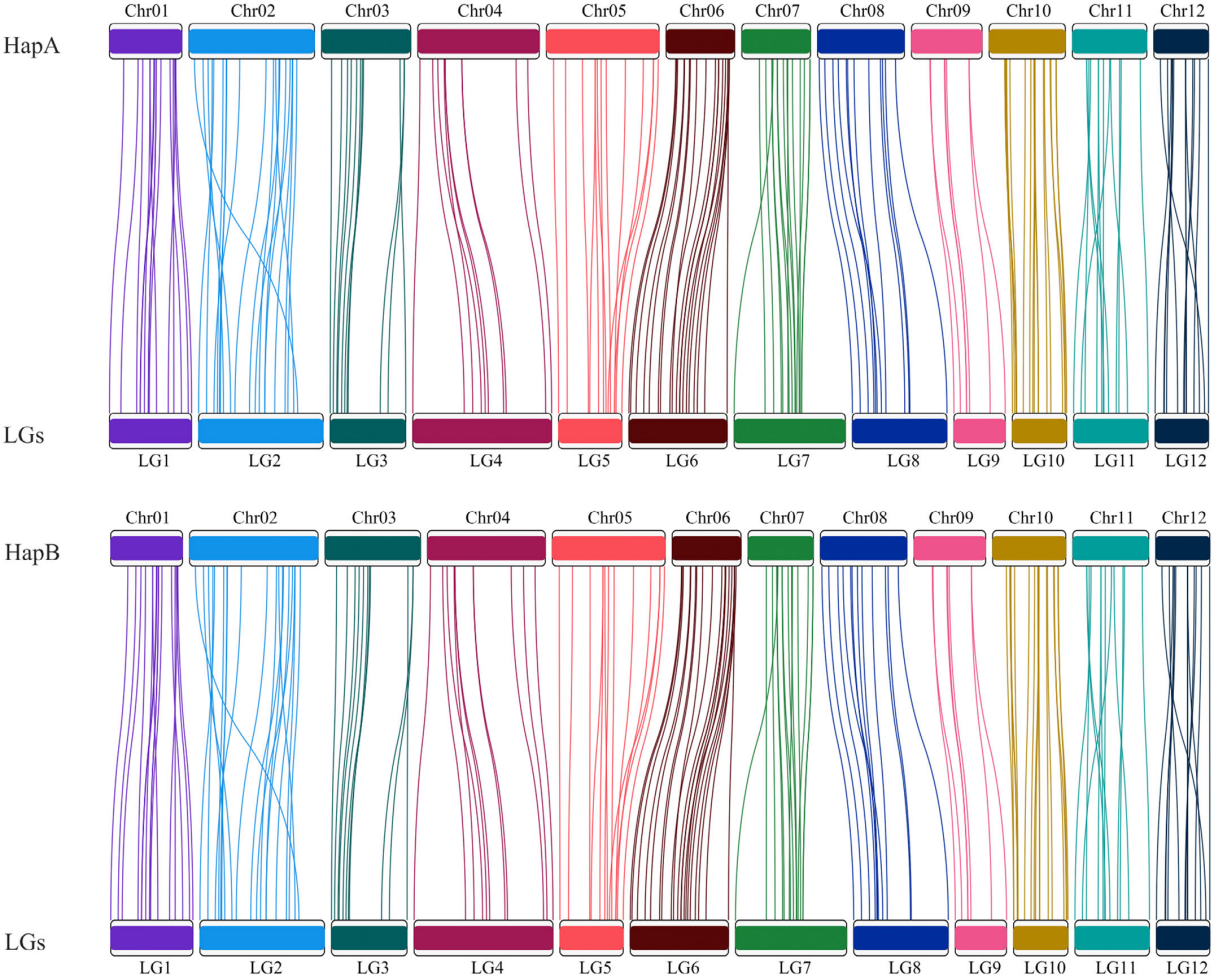
Comparison of the *Quercus alba* genetic map to the *Q. alba* genome assembly. The majority of markers from the genetic map (upper panel) map uniquely to the chromosomes in hapA and hapB assemblies (lower panel), visualized by colored lines.

A genetic map was constructed from the genotypes of 184 F1 progeny derived from an open‐pollinated mother tree (WO1; Fig. S4; Table S3) unrelated to the reference tree MM1. A total of 181 SNPs were organized into 12 linkage groups covering a total genetic distance of 731.5 cM, which is comparable with other *Quercus* genetic maps, including the 734 cM genetic map from *Q. robur*, the 764 cM genetic map from *Quercus petraea*, and the 652 cM genetic map from *Quercus rubra* (Bodénès *et al*., 2016; Konar *et al*., 2017; Table S4). The longest and shortest linkage groups were LG2 (89.2 cM) and LG9 (36.7 cM), respectively. Of the 177 genetic map markers with available sequence data, 167 and 166 mapped uniquely to the hapA and hapB assemblies, respectively (Fig. 2). All markers from each linkage group mapped to the corresponding chromosome in each haplotypic assembly. Most markers were in the same order in the chromosomes as in the linkage map. Markers occurring in a different order on the genetic map compared with the genome assembly may be explained by a relatively low number of F1 progeny.

**Fig. 3:**
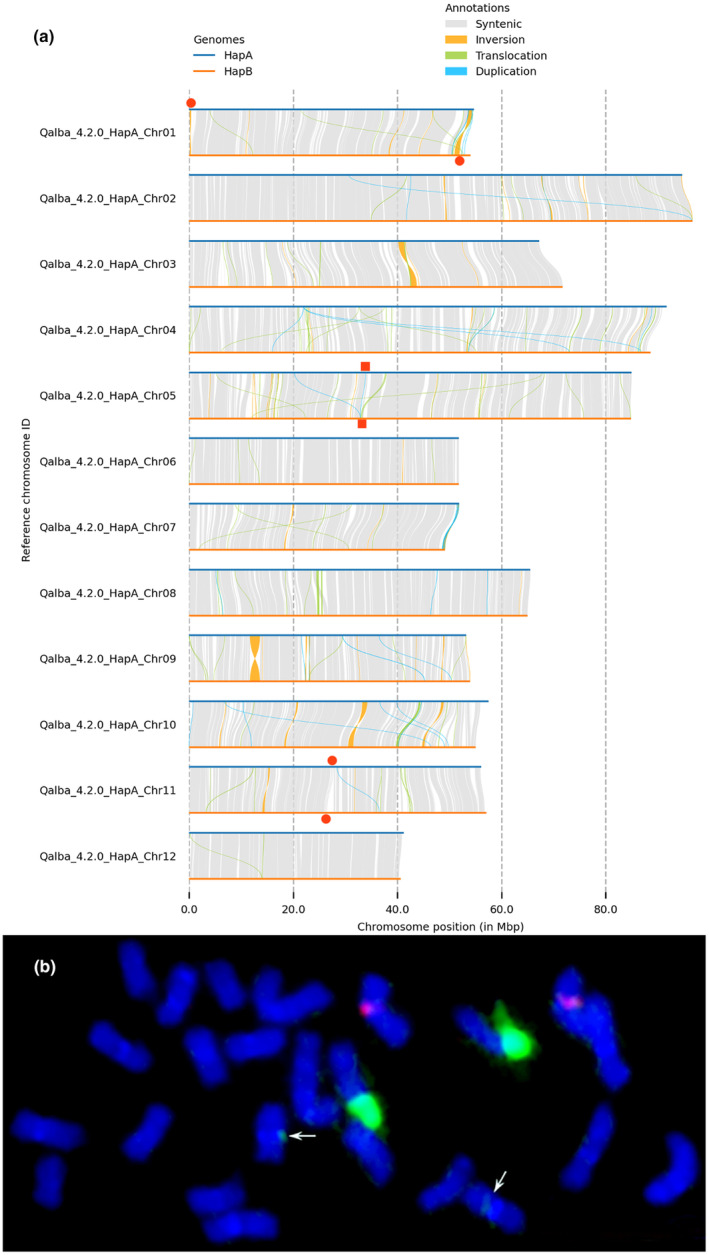
Genome structure of *Quercus alba* (a) Structural synteny between hapA and hapB of the *Q. alba* genome assembly. Two inversions are over 1 Mb: a 1.1 Mb inversion on Chromosome 3 and a 1.9 Mb inversion on Chromosome 9. The location of the 35S rRNA array is denoted by red circles, and the 5S rRNA arrays are denoted by red squares. (b) A metaphase chromosome spread with two pairs of 35S (green) and one pair of 5S (red) rRNA signals. Minor 35S rRNA signals are indicated by white arrows.

HapA and hapB were highly collinear across all 12 chromosomes. Based on sequence alignment and analysis by SyRI, 1283 syntenic blocks were identified, spanning 610 Mb in hapA and 620 Mb in hapB (Fig. 3). Based on these alignments, SyRI (Goel *et al*., 2019) identified 12 808 structural variants (SVs) of at least 100 bases (Table 2). Most SVs (96%) involved fewer than 10 000 bases, and only two SVs were over 1 Mb in length: a 1.1 Mb inversion on Chromosome 3 and a 1.9 Mb inversion on Chromosome 9. An additional 24 SVs were over 100 kb in length, including 14 inversions, five translocations, and one 155 kb section on Chromosome 1, which was absent in hapA. Because the PacBio HiFi reads averaged 20.8 kb in length and provided 45× coverage of the haploid genome, structural variants shorter than this average read length are likely to be real. Larger structural variants and those in highly repetitive regions may be real or may be artifacts of the assembly and scaffolding process.

**Table 2:** Structural variation identified between the two assembled haplotypes of *Quercus alba*.

	Total count	100 to < 1 kb	1 to < 10 kb	10 to < 100 kb	100 to < 1 Mb	> 1 Mb
Inversions	103	40	18	29	14	2
Translocations	1331	241	912	173	5	0
Insertions	4760	3621	1093	46	0	0
Deletions	4858	3730	1077	51	0	0
Duplicated regions	803	170	595	38	0	0
Inverted duplicates	652	138	431	83	0	0
Tandem repeat	15	12	3	0	0	0
Copy gain in hapB	134	61	54	16	3	0
Copy lost in hapB	152	84	49	18	1	0
All	12 808	8097	4232	454	23	2

### Structural and functional gene annotation

Repeats composed 58% and 59% of the hapA and hapB assemblies, respectively, with long terminal repeats (LTRs) as the largest class of elements at *c*. 28% of the genome in both haplotypes (Table S5). The unplaced scaffolds had a higher percentage of bases identified as repetitive than the chromosomes: 67% vs 57% for hapA and 82% vs 58% for hapB, suggesting that repetitive sequences may have interfered with the assembly process (Tørresen *et al*., 2019; Giani *et al*., 2020). The LAI (LTR Assembly Index), a metric of assembly quality based on completeness of LTRs (Ou *et al*., 2018), was 23.3 for hapA and 22.9 for hapB. These scores are in the top 10% of LAI scores of 103 high‐quality plant genomes as assessed in Ou *et al*. (2018), indicating high assembly quality through repetitive regions for both white oak haplotypes. Categories of repeat types found in hapA and hapB were similar, except that the DNA transposon Tc1/mariner superfamily was found to constitute only 0.08% of hapA but made up 1.43% of hapB (Notes S1; Table S6).

Protein‐coding gene annotation identified 42 955 genes in hapA and 42 412 genes in hapB. Over 99% of genes were on chromosome scaffolds. Based on structural variants identified between the haplotypes, we found that 2365 gene bodies (5.5% of genes) overlapped with an SV, and of those, 1374 (3% of genes) had an exonic sequence overlapped by an SV. The most common type of SV to overlap a gene was an inversion (803 genes) with insertions as the second most prevalent type (554 genes). Functional annotations were found for 82% of genes through sequence similarity to databases of proteins, metabolic pathways, and gene families (Zenodo, 10.5281/zenodo.14736109). Sixteen samples of RNA from four different white oak trees, including four emerging leaf samples from the MM1 tree, were used for annotation and then remapped to the annotated genome. Across samples, 81–91% of reads mapped uniquely, while 3–5% mapped to multiple locations. There were 29 594 genes in hapA that had at least one mapped RNASeq read and 23 639 had a TPM (transcript reads per kilobase of gene space per million) > 0.5. Tissues differed in number of genes expressed, from 17 582 genes in emerging leaves to 24 798 genes in the emerging radical apex of a germinating acorn (Table S7).

Several rRNA gene arrays were annotated in the genome by sequence similarity (Fig. 3a). A single 5S rRNA array was found on Chromosome 5 at 33.8 Mb in hapA (32.9 Mb in hapB). Two 35S rRNA arrays were found. One 35S rRNA array was located on Chromosome 1 at 160 kb in hapA, but found at the opposite end of Chromosome 1 in hapB (50–53 Mb). This is likely an assembly artifact due to the very highly repetitive nature of the rDNA and nearby telomeric repeats, but it is not clear from the Hi‐C evidence, which telomeric end is the correct location, so the haplotype assemblies were not altered. The second 35S array was found on Chromosome 11 at 27.4 Mb in hapA (26.2 Mb in hapB). To confirm rRNA arrays, FISH was conducted and revealed one 5S locus and one 35S locus consistently, with an additional, possibly minor, 35S locus observed rarely (i.e. not in every metaphase; Figs 3b, S5). These sites are located on three different chromosome pairs, which agrees with the sequence‐based analysis. The 5S and 35S rRNA sites appeared to be colocalized with AT‐rich heterochromatic bands (Fig. S5).

An initial assessment of annotated genes with nucleotide binding and leucine‐rich repeat (NLR) domains yielded 111 genes in hapA and 152 genes in hapB (Table 3). As NLR genes are known to be difficult to annotate and biased by repetitive element masking (Bayer *et al*., 2018), we then ran the full genomic sequence without masking through the FindPlantNLRs pipeline (Chen *et al*., 2023), which yielded 1042 and 1056 NLR genes for hap A and B, respectively. Based on additional domains, the genes were further classified into four main categories (Table 3). RxNL (Rx N‐terminal domain‐NLR) were the most common, followed by TNL (Toll/interleukin‐1 receptor‐type (TIR)‐NLR), CNL (Coiled coil (CC)‐NLR), and RNL (Resistance to Powdery Mildew 8 (RPW8)‐NLR) as the smallest gene category. Over 85% of the newly annotated R genes overlapped by at least 10% of their length with an annotated repetitive element, suggesting that repeat masking may contribute to their absence from the original gene annotation. All categories of R genes were highly clustered, with 84% of RxNL, 79% of TNL, 70% of CNL and 77% of RNLs found in a localized cluster of genes in the same category (Fig. S6).

**Table 3:** R gene domains and R gene classifications identified between the two assembly haplotypes of *Quercus alba*.

	R genes in HapA	No. of clusters in HapA	% of R genes in clusters in HapA	Largest cluster in HapA	Genes in HapB	No. of clusters in HapB	% of R genes in clusters in HapB	Largest cluster in HapB
RxNL	434	67	84	21	421	64	85	28
TNL	327	60	79	16	348	61	79	18
CNL	95	21	74	6	90	17	67	10
RNL	20	2	75	9	28	3	79	14

### Clustering, PCA, and population genetics

**Fig. 4:**
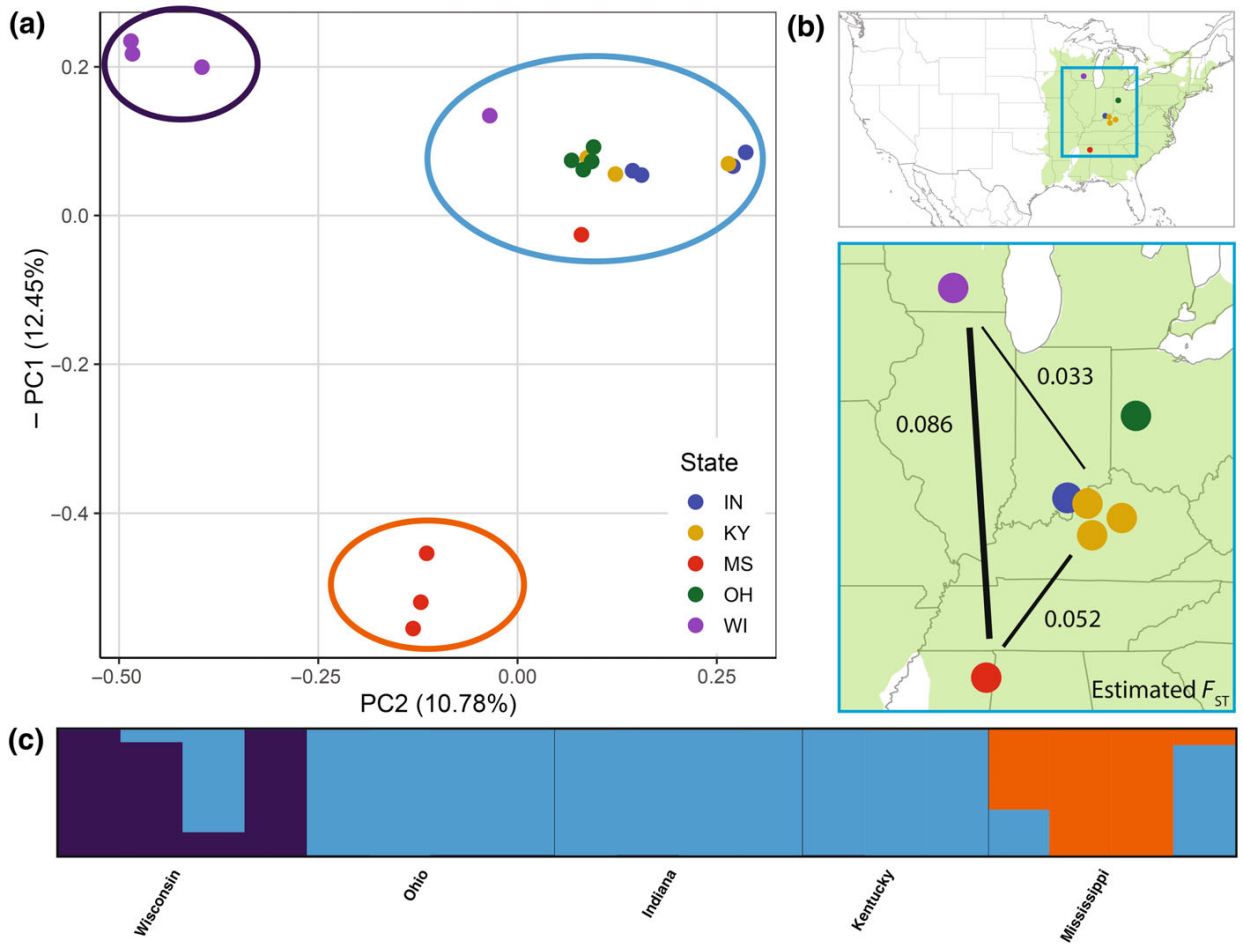
Population structure within *Quercus alba*. (a) Principal component analysis of 19 *Q. alba* individuals. The inverse of PC 1 is plotted on the *y*‐axis and PC 2 is plotted on the *x*‐axis. Colored circles around clusters correspond to the color scheme of (c) and denote the major ancestry of those individuals in Structure analyses for *K* = 3. (b) Map of sample provenances colored by state with the range of *Q. alba* shown in green. Numbers correspond to *F*_ST_ (Reich–Patterson estimator) between groups of samples from Wisconsin, MS and a combined group from Indiana, Ohio, and Kentucky. (c) Structure result for a typical replicate with *K* = 3 for the 19 individuals of *Q. alba* from five US states.

We performed whole genome shotgun sequencing of 18 *Q. alba* individuals and analyzed these data alongside those of our reference individual (Table S1). Our filtered dataset for the 19 *Q. alba* samples consisted of 50 461 765 SNPs, 7.5% of which had three or more alleles (including single nucleotide deletions). We summarized our Structure results with CLUMPAK, which revealed high consistency among replicate runs for values of *K* = 1, 2, and 3, with all 10 replicates clustering into a single mode per value of *K* (Fig. S7). PCA revealed that the first two principal component axes strongly correlated with the geographic origin of samples (Fig. 4). The clustering pattern of individuals in the PCA was also qualitatively consistent with Structure results at *K* = 3. Pairwise *F*_ST_ with the Reich–Patterson (2009) estimator was greatest between the Wisconsin and Mississippi populations (*F*_ST_ = 0.086; Table S8). The estimated value of π (nucleotide diversity) was 1.2% when considering all 19 *Q. alba* individuals as a single population (Table S9).

### Phylogenomic analyses

**Fig. 5:**
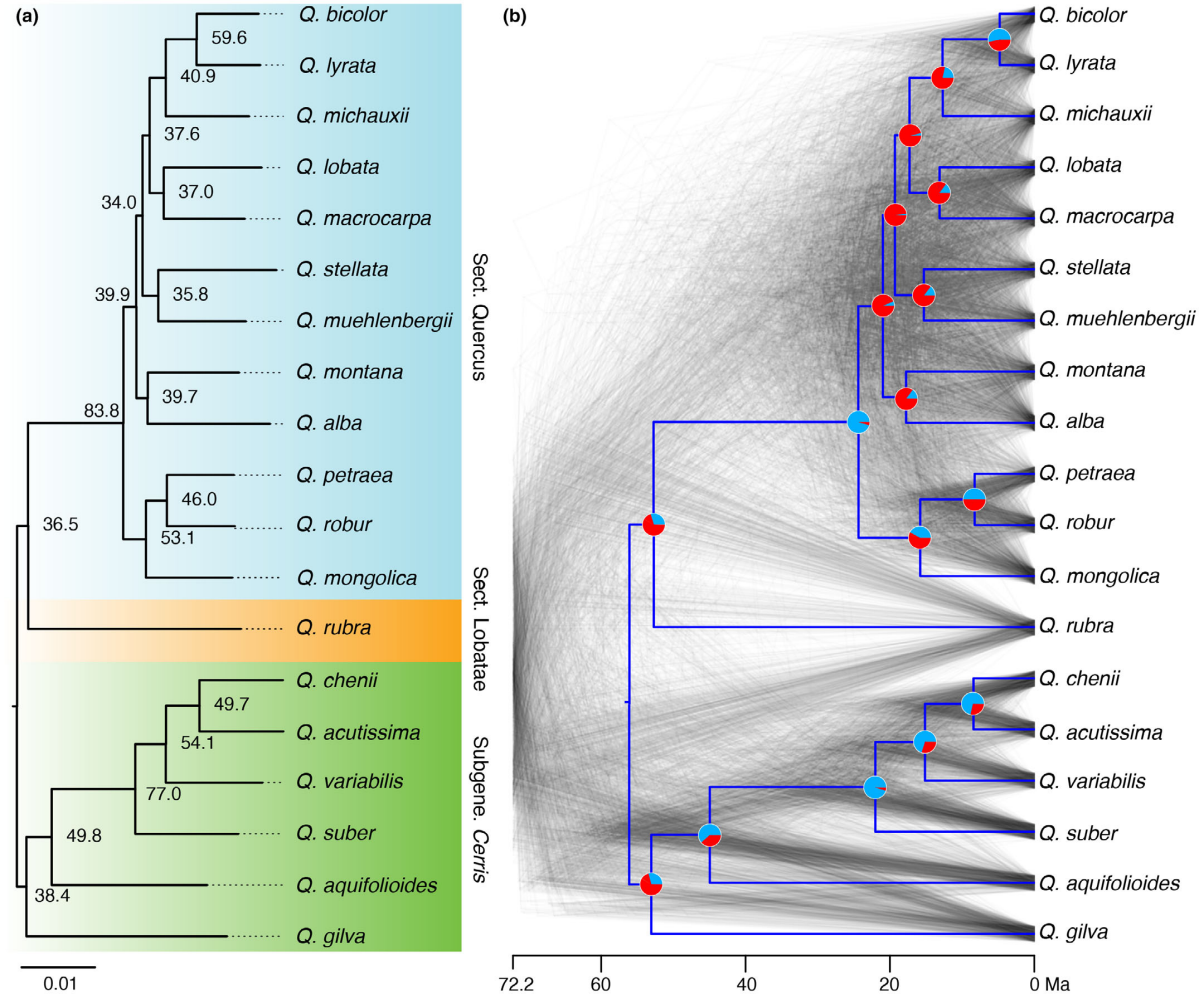
Phylogenetic relationships among species of *Quercus*. (a) Phylogenetic tree estimated with maximum likelihood from a concatenated alignment of genome sequences from 19 *Quercus* species and rooted on an individual of *Lithocarpus* (not depicted). Node values are site concordance factors and all species relationships received 100% ultrafast bootstrap support. Branch lengths are in units of estimated substitutions per site. (b) The time‐calibrated species tree (blue) and a cloudogram based on 1000 time‐calibrated window trees. Pie charts at nodes indicate the proportion of 12 048 window trees that are concordant (light blue) or discordant (red) with the species tree.

We produced whole genome shotgun sequencing reads for individuals of seven North American white oak species, which were analyzed along with public short‐read data from an additional 15 *Quercus* species and *Lithocarpus longipedicellatus* (Table S1). Our final phylogenomic dataset consisted of 327 242 758 aligned sites that were not masked in all samples. Based on our maximum likelihood tree, all individuals of *Q. alba* formed a clade that was sister to *Quercus montana* (Fig. S8). All individuals from North American white oak species formed a clade, which was sister to a clade composed of *Q. robur*, *Q. petraea*, and *Q. mongolica*, the other three white oak species in our sampling (Fig. 5). Section *Lobatae*, represented by *Q. rubra*, was sister to the white oak clade.

Both site concordance factor (sCF) and gene concordance factor (gCF) values indicated extensive phylogenomic discordance for many relationships, especially within the more intensively sampled white oak clade. Within this clade, there are several branches that were supported by fewer than 10% of genomic windows. There was no tree for any 5‐kb window that was completely concordant with the inferred species trees. The Astral species tree was similar in topology to the maximum likelihood tree, except for several relationships within the white oak clade, which were subtended by extremely short branches (Fig. S9). In the chloroplast and mitochondrial trees, *Q. alba* was not monophyletic, and both trees were characterized by short internal branches within section *Quercus* and by widespread phylogenetic conflict with one another (Figs S10–S12).

There were 4417 437 sites (singletons excluded) inferred to be variable among *Q. alba* individuals; of these, 57.7% of sites were inferred to be variable among other white oak species (Fig. S8; Table S10). When singletons were included, there were 16 388 119 variable sites within *Q. alba*, with 43.6% of those also variable among other white oak species. Correcting for this ancestral variation resulted in divergence time estimates that were up to 9.9 million years (Myr) closer to present than analyses that used uncorrected branch lengths (Fig. S13).

### Genomic architecture across the *Quercus* clade

**Fig. 6:**
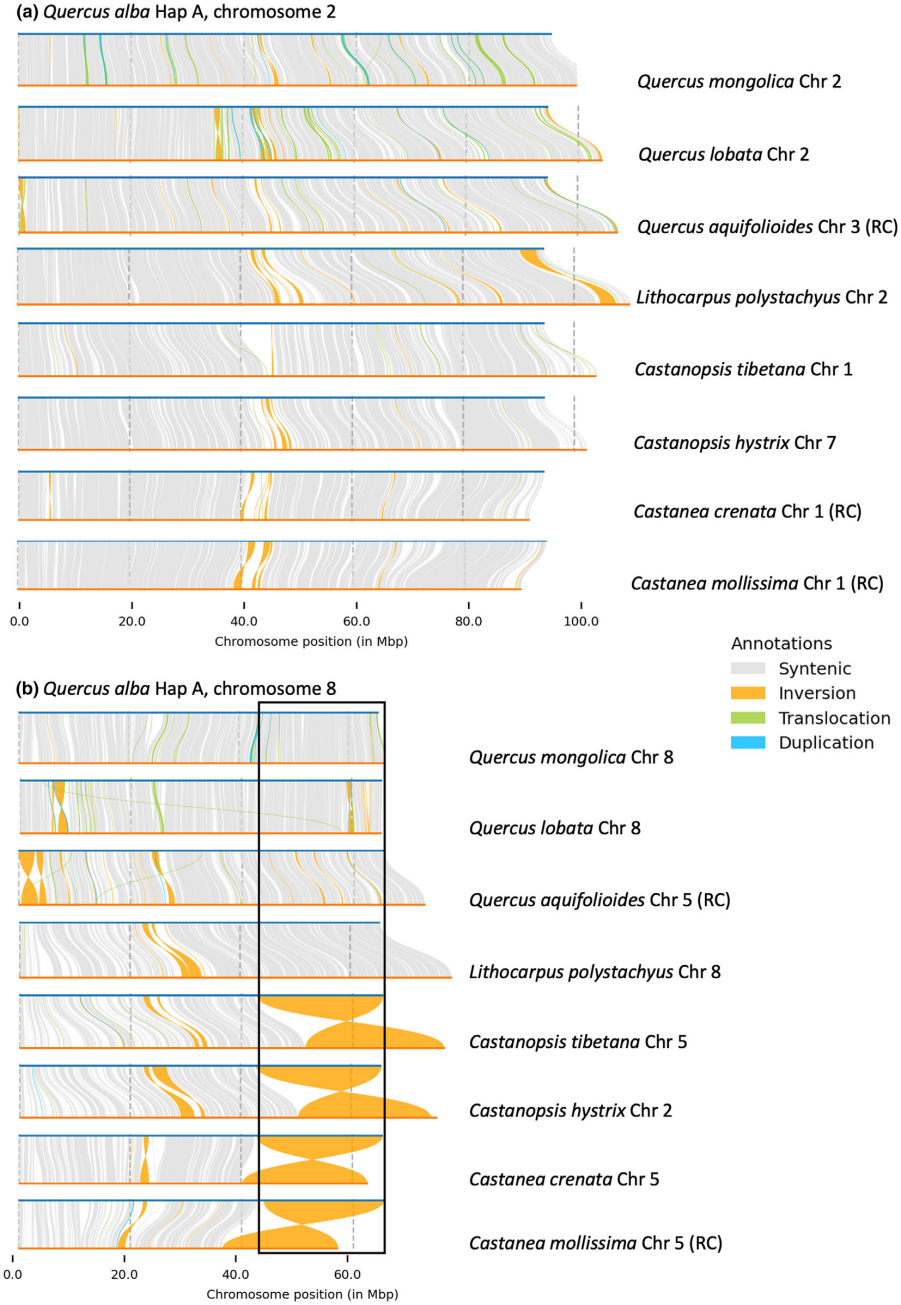
Structural synteny of *Quercus alba* hapA (top bars, blue) vs homologous chromosomes (bottom bars, orange) of eight other Fagaceae species. Data are visualized in an order consistent with the phylogeny. (a) An example of a typical comparison (Chromosome 2), showing strong collinearity overall with small structural rearrangements. (b) Chromosome 8, for which investigated members of *Quercus* and *Lithocarpus* share a large inversion relative to other Fagaceae. Comparisons of the remaining chromosomes across the same species and additional *Quercus* genomes are available in Supporting Information Figs S14 and S15.

Of the *Quercus* species included in our phylogenetic analysis, eight in addition to *Q. alba* have available chromosome‐scale genomes, providing an opportunity to examine the evolution of chromosome structure across the genus (Fig. S1; Table S11). Using *Q. alba* as the reference, the genomes were aligned and assessed for structural variation, revealing that they share strong chromosome‐to‐chromosome syntenic structure (Figs 6a, S14). To examine whether the chromosome synteny is preserved in other genera of the Fagaceae, five species with chromosome‐scale genomes from three other genera were also examined for syntenic structure. A major structural variant, a 22 Mb inversion at the end of Chromosome 8, was found to be nearly identically located in *Castanopsis* and *Castanea* species (Fig. 6b) but was not found in any *Quercus* species or *Lithocarpus polystachyus*. The other 11 chromosomes were largely syntenic.

**Fig. 7:**
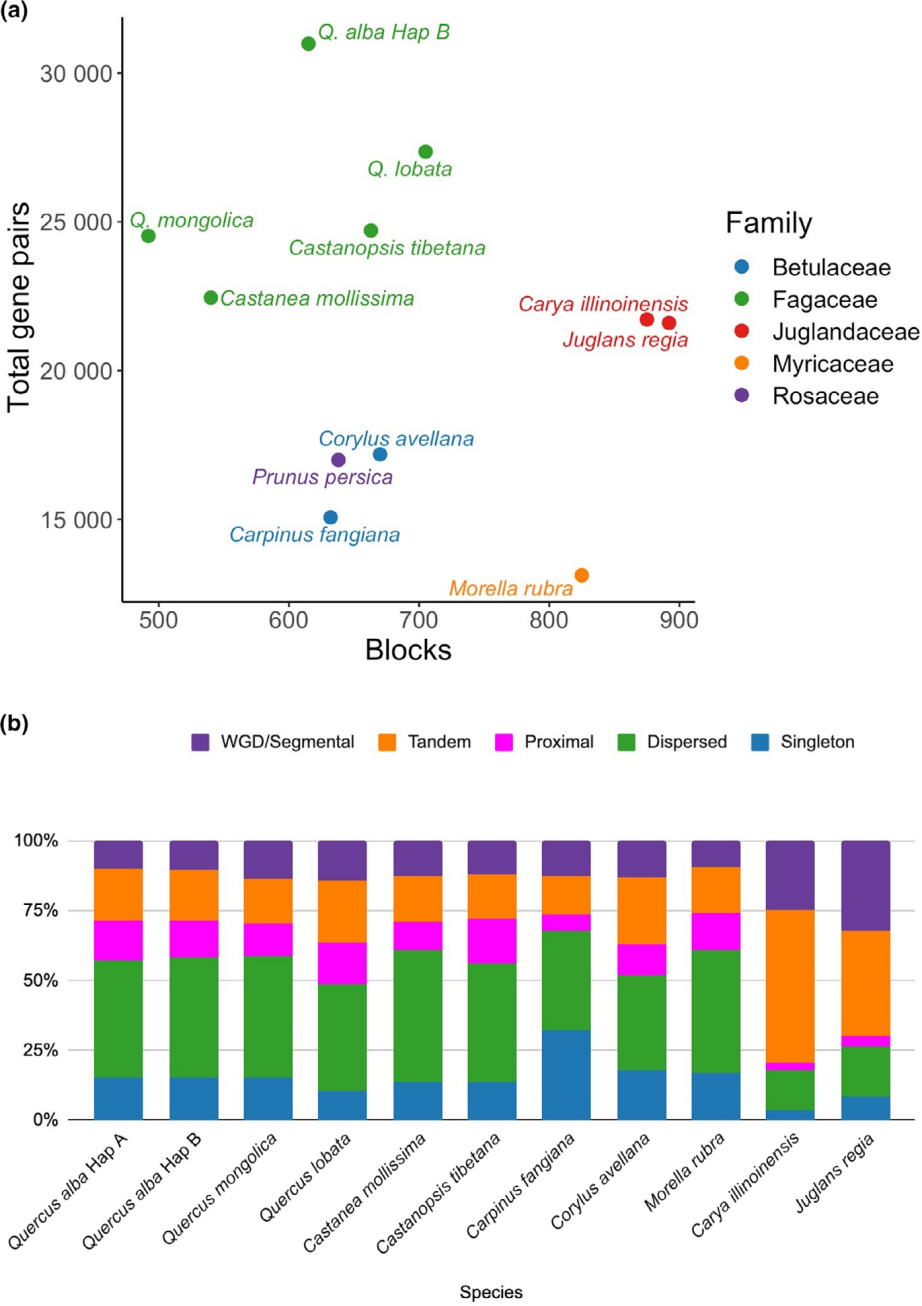
Comparative analysis of genome collinearity and gene duplications between *Quercus alba* and tree species from four additional rosid families. (a) Comparison between the number of blocks of collinear genes and the total number of collinear genes identified. (b) Relative percentage of gene pairs within a genome generated by whole genome duplications, tandem duplications, proximal duplications, dispersed duplications, or no duplication, that is singleton genes.

Examining species outside Fagaceae, many major chromosomal differences are evident, and chromosomal segments that can be directly mapped based on sequence similarity are shorter. However, despite nucleotide divergence, gene collinearity is often conserved in large blocks (Figs 7a, S14, S15). To further characterize the nature of gene duplication across this same set of tree species, we used MCScanX to classify genes as originating from whole genome duplications, tandem duplications, proximal duplications, dispersed duplications, or single copy genes (i.e. no duplication). Interestingly, all species in the Fagales share very similar relative percentages of these categories, with most genes classified in the dispersed duplicates category (Fig. 7b).

### Gene family evolution among *Quercus*

**Fig. 8:**
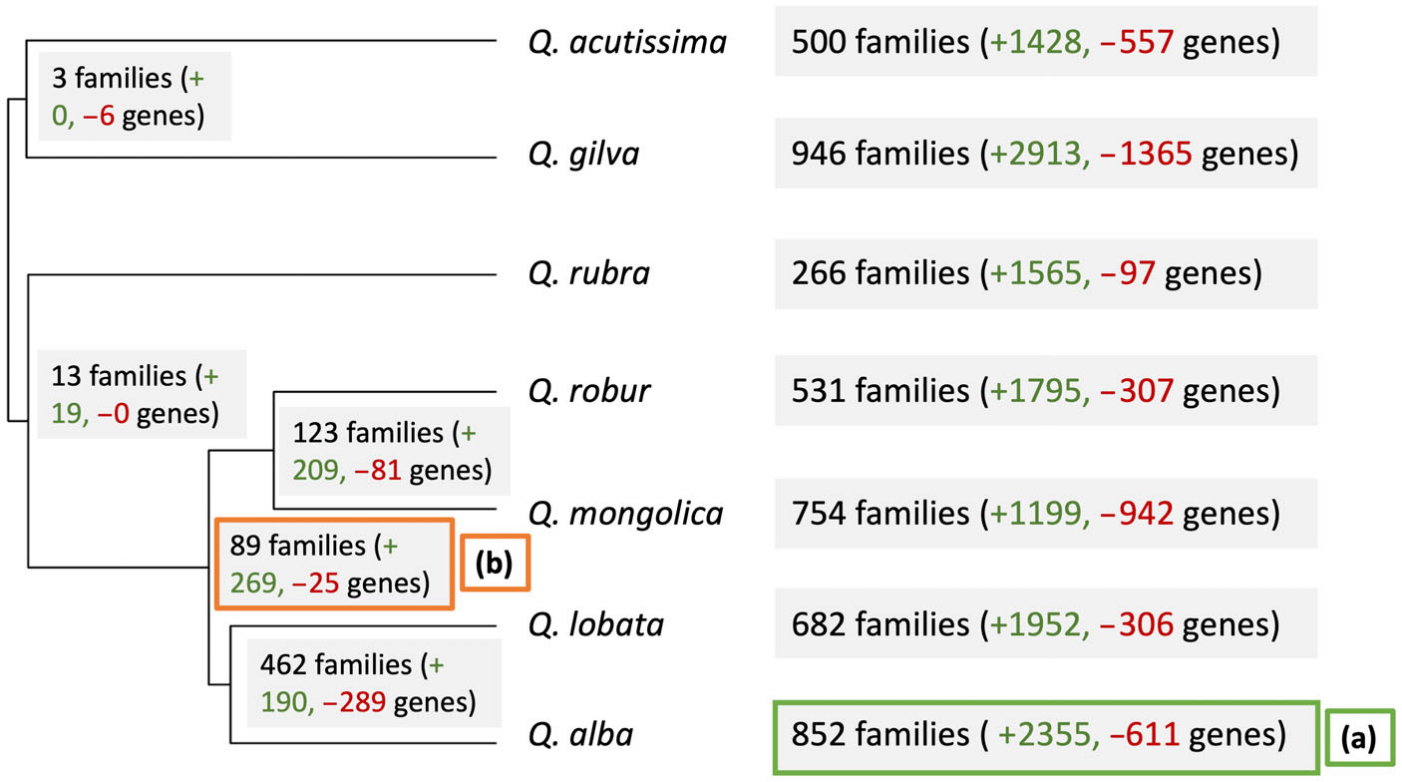
Cafe detected gene families with significantly accelerated gene gain or loss on each branch of the phylogenetic tree (grey boxes). Gene Ontology enrichment was conducted to functionally profile the rapidly changing gene families in the *Quercus alba* genome since its most recent ancestor with *Quercus lobata* (a) and for the white oak clade (section *Quercus*) after branching from the rest of the *Quercus* genus (b).

The combination of fully annotated genomes and an ultrametric phylogeny of *Quercus* enabled examination of the evolution of gene families across the genus. Using all annotated protein‐coding genes with chromosomal placement from seven *Quercus* species, OrthoFinder identified 30 270 total orthogroups (mean size of 7 genes) and 13 623 orthogroups with all species present. Cafe was used to model gene family evolution and to detect families with significantly accelerated gene gain or loss on each branch of the phylogenetic tree. Cafe identified 852 gene families in *Q. alba* that have rapidly evolved in gene copy number since the last common ancestor with *Q. lobata* (*P* < 0.05), reflecting a gain of 2355 genes and a loss of 611 genes (Fig. 8a). Based on Gene Ontology (GO) enrichment analysis (*P* < 0.05) of these rapidly evolving families, of the top 20 significantly enriched biological process terms, seven of those terms related to external stimulus and stress response (Table S12). Two additional terms relate to growth regulation. We identified 89 significantly changing gene families along the branch subtending *Quercus* section *Quercus*, with 269 genes gained and 25 genes lost (Fig. 8b). Of the top 20 GO terms enriched in these rapidly evolving families, six were related to defense (Table S13).

**Fig. 9:**
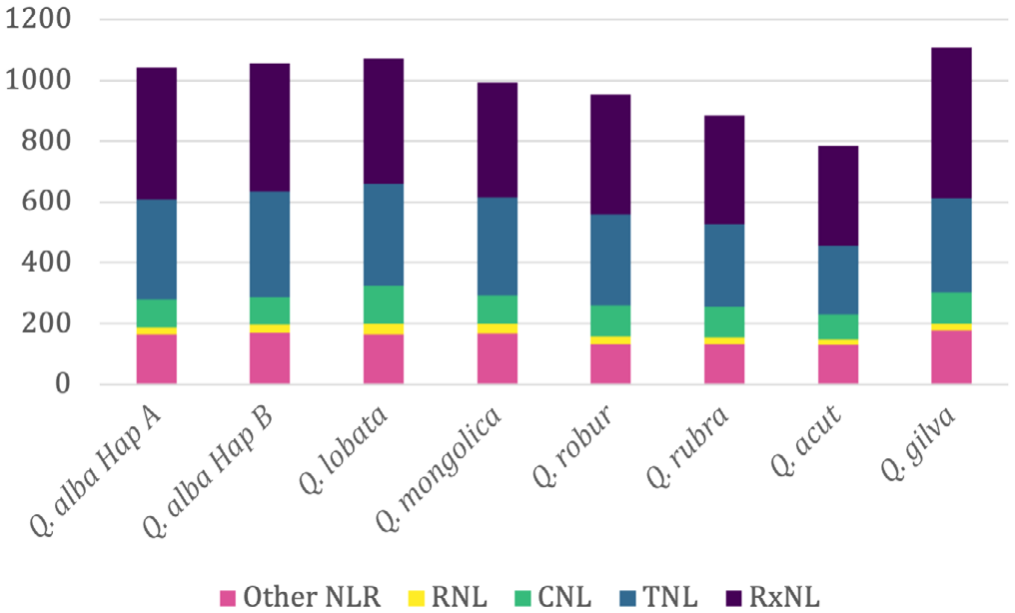
Number of nucleotide binding and leucine‐rich repeat (NLR) genes (*y*‐axis) that were recovered from *Quercus* genomes in four specific categories, RNL, CNL, TNL, and RxNL, plus unclassified NLRs.

**Fig. 10:**
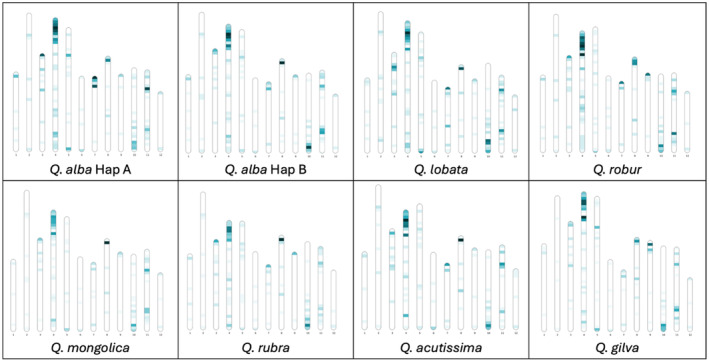
Locations of RxNL genes on the chromosomes of *Quercus* genomes. Each of the 12 chromosomes are oriented and ordered by synteny to the *Quercus alba* chromosomes (Supporting Information Table S15). Density of RxNL genes is visualized as a heat map in 2 Mb segments, with lighter color indicating fewer genes and darker color indicating more genes. RxNL gene clusters are consistently found at the ends of Chromosomes 4, 8, and 10. Chromosomal heatmaps for RNL, CNL and TNL genes show similar patterns of conserved cluster locations (Figs S16–S18).

To further examine the evolution of defense response genes without bias induced by differing annotation pipelines, we ran the same R gene identification pipeline used for *Q. alba* on seven unannotated, unmasked *Quercus* genomes (Fig. 9). The overall number of NLR genes varied from 785 in *Quercus acutissima* to 1109 in *Quercus gilva*. *Quercus acutissima* consistently had the lowest number of annotated genes per category (RxNL, TNL, CNL, and RNL). All species had the majority of NLRs in the RxNL category followed by TNLs and CNLs, with RNLs being the least abundant category (Table S14). For all categories of R genes, these genes tended to occur in clusters. Although the size of these gene clusters varied between species, they were generally conserved in the same general syntenic locations on the 12 chromosomes (Figs 10, S16–S18; Table S14). RxNLs had large clusters across many chromosomes, with the largest cluster ranging from a 34 gene cluster in *Q. mongolica* on Chromosome 8 to a 14 gene cluster on Chromosome 4 in *Q. lobata* (Fig. 10). The largest CNLs clusters ranged from six genes in *Q. alba* hapA and *Q. mongolica* to 21 genes in *Q. gilva*. Large CNL clusters are found on Chromosomes 3, 7 and 8 in most species. For TNLs, the largest cluster per species ranged from 20 in *Q. gilva* to nine in *Q. lobata* with the largest clusters consistently found on Chromosomes 3, 7, and 9. RNLs occur on all chromosomes except 7, 10, 11, and 12, and the largest cluster is always found on Chromosome 6.

## Discussion

### The *Q. alba* genome

We generated a haplotype‐resolved and chromosome‐scale *Q. alba* reference genome that will support new genetic and genomic research in forest trees (Neale & Kremer, 2011; Plomion *et al*., 2016b). Genomics can be a key component of creating a sustainable supply of white oaks for ecosystem management, forest restoration, and wood products such as lumber, veneer, and barrels for aging wine and spirits (Grattapaglia *et al*., 2009; Wheeler *et al*., 2015). There are currently active tree improvement programs conducting progeny trials and establishing seed orchards to supply genetically improved white oak acorns for nursery production (Schlarbaum, 1993, 2000, 2024; Dewald *et al*., 2023). These programs currently select for heritable traits such as rapid early growth and apical dominant architecture but are limited in progress by multidecade generation times and huge space requirements. Leveraging the *Q. alba* reference genome to map traits and to establish molecular screening approaches for early selection of high performing material or to implement a breeding program with genomic selection promises to rapidly advance the quantity and quality of white oak germplasm (Wheeler *et al*., 2015). The white oak genome will also open avenues to study genes underpinning traits characterized in other tree species, including wood‐quality traits such as lignocellulose composition and optimization of flavor expression in oak barrels used for spirit aging (Gollihue *et al*., 2018, 2021; Grattapaglia *et al*., 2018).

We confirmed the completeness and accuracy of both genome assembly haplotypes with Busco and LAI scores as well as a new genetic linkage map. The haplotypes have contig N50s of 8.3 and 8.9 Mb, commensurate with the highest quality recent plant genome assemblies (Kong *et al*., 2023), with 97% of bases scaffolded into chromosomes (Table 1). In agreement with other *Quercus* genomes (Ai *et al*., 2022; Sork *et al*., 2022), cytology (Friesner, 1930), and *C*‐value estimates (Bai *et al*., 2012), each haplotype has 12 chromosomes and is *c*. 793 Mb in length.

Despite the growing number of publicly available oak genomes, few are haplotype‐resolved and offer the ability to assess SVs within a single individual's genome. The two *Q. alba* haplotypes have not only highly similar overall structures, as expected, but also extensive structural variation (Fig. 3). We identified over 12 000 structural variants, with insertions and deletions as the most common type, followed by duplications and translocations (Table 2). The number of SVs in *Q. alba* is similar to those reported in the haplotype‐resolved genome of *Quercus variabilis* (103 vs 64 inversions; 1331 vs 1600 translocations) and much lower than the number reported in *Quercus glauca* (103 vs 1136 inversions; 1331 vs 10 950 translocations; L. Wang *et al*., 2023; W. Wang *et al*., 2023; Luo *et al*., 2024). SVs are generally deleterious but have also been identified as drivers of adaptation and the causative mutations underlying phenotypic differences in woody plants (Zhou *et al*., 2019; Guo *et al*., 2020; Hämälä *et al*., 2021). Supporting their potential importance in the white oak genome, we found that 5.5% of gene bodies overlapped SVs and 3% of genes overlap SVs in exonic regions. This is likely an underestimate of *Q. alba* genes impacted by SVs throughout the species as this study leveraged SVs found only within a single individual. Indeed, further range‐wide evaluation of SVs in *Q. alba* is needed to carefully evaluate their adaptive importance.

It is still unclear whether the large structural variants we observed originated from assembly or scaffolding errors, particularly for SVs that are longer than individual PacBio HiFi reads (Yuan *et al*., 2021). While the single 5S rRNA array and one of two 35S rRNA arrays were placed in the same chromosomal locations between hapA and hapB, the other 35S rRNA array was placed on opposite ends of Chromosome 1 (Fig. 3). rRNA arrays have been found particularly difficult to assemble in other species as well (Navrátilová *et al*., 2022; Huff *et al*., 2023), and we posit that due to its location in the highly repetitive telomeric region of Chromosome 1, our methods were unable to consistently place one 35S rRNA array. The confirmation of large structural variants, particularly those in repetitive regions, will require additional investigation.

### The evolution of gene family size across the *Quercus* phylogeny

Disease resistance genes (R genes), also referred to as pathogen recognition genes encompass multiple categories of defense genes with conserved domain patterns essential to pathogen recognition and defense initiation (Yue *et al*., 2012; Fischer *et al*., 2016; Liu *et al*., 2017). Genes functioning in defense has been identified as some of the most rapidly evolving gene families in plants with signatures of both purifying and positive selection (Fischer *et al*., 2016; Zheng *et al*., 2016). We analyzed rapidly changing gene families along two branches of our phylogenetic tree: the terminal branch leading to *Q. alba* and the branch subtending section *Quercus*. In both cases, defense‐related genes were strongly enriched, suggesting that, despite long generation times, R gene families appear to be capable of rapid evolution in tree species. It should be noted that Cafe is not able to model gene tree discordance, which may lead to increased false‐positive rates of rapidly evolving families (Neafsey *et al*., 2015; Mendes & Hahn, 2016).

After the initial assembly of the first oak reference genome, *Q. robur*, Plomion *et al*. (2018) noted an enrichment of R genes compared with other plant species. This was echoed by Sork *et al*. (2022), who further noted patterns of tandem and proximal duplication in R gene evolution in *Q. lobata*. By contrast, Ai *et al*. (2022) reported a notable decrease in R genes in *Q. mongolica* (Ai *et al*., 2022), identifying 302 NBS genes in comparison with 1019 in *Q. robur* and 1171 in *Q. lobata*. However, these published *Quercus* reference genomes used different repeat annotation and gene identification pipelines. As previous studies have found a bias in R gene annotation due to different annotation approaches (Bayer *et al*., 2018), we identified and annotated R genes with a *de novo* pipeline, FindPlantNLRs (Chen *et al*., 2023), using unmasked and unannotated genomes from *Q. alba*, and seven additional *Quercus* species. With this approach, we found that R genes vary in number considerably less across species than previously reported. For example, we found *Q. mongolica* had 994 NLR genes, very similar in overall quantity to the 953 in *Q. robur*, 1070 in *Q. lobata*, and 1042 in *Q. alba* hapA. Categories of R genes and their cluster locations on chromosomes are largely conserved; however, individual counts of genes in each cluster varies between species. Our R gene annotation did not utilize transcriptome data, and future work verifying gene transcription and characterizing expression patterns in response to different stimuli is an important avenue of additional work to further understand the evolution of the R genes in oaks.

### Genomic diversity of *Q. alba*

We found evidence that *Q. alba* maintains high genetic diversity, though our sampling lacked representation from the western‐ and eastern‐most parts of the species' range. Thus, while our sampling precludes us from drawing range‐wide conclusions, our results do provide the first genome‐wide assessment of genetic diversity in *Q. alba* to date. Our overall estimate of nucleotide diversity (π) was 1.2%, which is similar to estimates from other broadly distributed oak species (Plomion *et al*., 2018). Notably, a high proportion of the overall genetic diversity of the species is shared among populations, with most individual populations having values of π *c*. 1% (Table S9). Our results also showed modest population structure within *Q. alba* (Fig. 4; Table S8). PCA revealed that, based on the first two principal components, samples were generally more genetically similar to those with a similar geographic origin. Structure analysis clustered samples in a similar way, with the Wisconsin and Mississippi populations largely forming distinct clusters and the remaining samples clustering together. This pattern of clustering is also evident in our phylogenetic results (Fig. S8). While there is some evidence from provenance trials that some populations of white oak may be locally adapted, including exhibiting differences in leaf phenology (Thomas *et al*., 2024), there remains much to be learned about genetic differentiation across the species range.

### Shared variation among oak species and implications for divergence time estimation in oaks

We found that there are millions of shared variable sites among white oak species, which has important implications for phylogenetic analysis and divergence time estimation. By some metrics, as many as 57.7% of variable sites within *Q. alba* were also variable among other white oak species (Fig. S8). Such a result suggests that much of this variation has been shared since their common ancestor. When analyzed in a typical phylogenetic framework, this shared variation is ignored, and when different alleles are sampled in different individuals, these may appear as differences between populations or species. This effect was apparent in our results: In many cases, the inferred genetic divergence between individuals within *Q. alba* was nearly as high as between several species pairs (Fig. S8). An important downstream effect of mistaking ancestral variation for differences that have accumulated since speciation is the inference of erroneously long branch lengths, which has the effect of ‘pushing back’ estimated divergence times (Edwards & Beerli, 2000). To account for this effect, we used our estimate of π from *Q. alba* to correct branch lengths and to account for this ancestral diversity, which for many nodes resulted in divergence times that were 8–10 Myr nearer to the present than when using uncorrected branch lengths analyzed with the same dating methods (Fig. S13).

### The history of the white oak clade is characterized by extensive phylogenomic conflict

Our results using whole genome alignments suggest a different phylogenetic history for some taxa compared with a recent, broadly sampled phylogenetic analysis based on RAD‐Seq data (Hipp *et al*., 2020), though our results agree for many relationships (Notes S2). There are several factors that likely contribute to these observed differences, including differing approaches to sampling and sequencing. Our phylogenetic analysis included far fewer taxa than that of Hipp *et al*. (2020), but many more aligned sites than can be recovered with RAD‐Seq. Oaks are also often reported to hybridize (e.g. Leroy *et al*., 2017; Hipp *et al*., 2019; Degen *et al*., 2023), and geographic differences in patterns of introgression as well as pervasive phylogenetic conflict may have contributed to differences in our phylogenetic results. While detailed investigation of patterns of hybridization and introgression is beyond the scope of this work, we found high phylogenetic discordance across the white oak clade and that a large proportion of sites that were variable within *Q. alba* were also variable among other species of white oaks (Fig. S7). These findings could be due, in part, to introgression as well as incomplete lineage sorting following rapid radiations in the clade. Hardin (1975) described morphological evidence of hybridization between *Q. alba* and nine other species in the white oak clade, which includes *c*. 15 other species in eastern North America. Hybridization followed by backcrossing can facilitate the transfer of adaptive alleles across species boundaries, as has been suggested by recent evidence in European white oaks (Leroy *et al*., 2020). Future work should address the role of introgression in the white oak clade using whole genome data.

## Competing interests

None declared.

## Author contributions

DAL, MES, SES, TZ, MWH, JEC, AGA, SD and CDN designed the research. DAL, MES, BK, SF, JS, NI‐F, SES, AH, ECS, TZ, JEC and CDN performed the research. DAL, MES, BK, SF, JS, AT, NI‐F, ASA, ECS, TZ, MWH and JEC contributed to data analysis, collection and/or interpretation. DAL and MES wrote the manuscript with contributions from BK and NI‐F. All authors reviewed and approved the manuscript. MES and DAL contributed equally to this work and share co‐first authorship.

## Disclaimer

The New Phytologist Foundation remains neutral with regard to jurisdictional claims in maps and in any institutional affiliations.

## Supporting information


**Fig. S1** Summary of taxa included in each comparative genomics analysis.
**Fig. S2** The white oak (MM1) chloroplast genome.
**Fig. S3** Annotated, circularized draft assembly of the *Quercus alba* (MM1) mitochondrial genome.
**Fig. S4** Female WO1 map composed of 181 SNP markers.
**Fig. S5** Fluorescence *in situ* hybridization of white oak chromosome spreads reveals two pairs of 35S (green) and one pair of 5S (red) rRNA signals.
**Fig. S6** Visualization of R genes on *Quercus alba* Hap A and Hap B chromosomes.
**Fig. S7** Major modes recovered with CLUMPAK for Structure results for *K* = 1–5.
**Fig. S8** Phylogenetic tree of *Quercus* including all sampled individuals of *Quercus alba* and results regarding shared variable sites.
**Fig. S9** Astral tree, generated from 12 081 gene trees based on 5 kb windows.
**Fig. S10** Chloroplast genome tree.
**Fig. S11** Mitochondrial genome tree.
**Fig. S12** Topological comparison between the chloroplast genome and mitochondrial genome trees.
**Fig. S13** Dated phylogenies.
**Fig. S14** Overview of structural synteny of *Quercus* genomes.
**Fig. S15** Detailed structural synteny.
**Fig. S16** Locations of TNL genes on the chromosomes of *Quercus* genomes.
**Fig. S17** Locations of CNL genes on the chromosomes of *Quercus* genomes.
**Fig. S18** Locations of RNL genes on the chromosomes of *Quercus* genomes.
**Methods S1** Supporting methods.
**Notes S1** Details of differences in Tc1/mariner family of repeats in HapA and HapB.
**Notes S2** Additional discussion of phylogenetic relationships.

**Table S1** Sample locations and NCBI data accession numbers for trees used for population genetics and phylogenetic analysis.
**Table S2** DNA and RNA sequence data statistics.
**Table S3** *Quercus alba* genetic map markers, locations, and sequences.
**Table S4** Genetic linkage maps of *Quercus robur* and *Quercus petrea* (Bodénès *et al*., 2016), *Quercus rubra* (Konar *et al*., 2017) and *Quercus alba* (this study).
**Table S5** Repeat profiles for the *Quercus alba* genome.
**Table S6** Tc1/Mariner superfamily profiles for the *Quercus alba* genome.
**Table S7** Genes expressed by tissue in *Quercus alba*.
**Table S8** *F*_ST_ values between *Quercus alba* populations.
**Table S9** Estimated nucleotide diversity (π) in *Quercus alba* populations.
**Table S10** Results from shared variable sites analysis among white oak species.
**Table S11** Structural variation of *Quercus alba* vs 10 Fagales genomes.
**Table S12** Gene Ontology terms enriched in rapidly evolving gene families in *Quercus alba* since its most recent common ancestor with *Quercus lobata*.
**Table S13** Gene Ontology terms enriched in rapidly evolving gene families in *Quercus* section *Quercus*.
**Table S14** Number of R genes and R gene clusters in eight *Quercus* genomes.
**Table S15** Order and orientation of *Quercus* genome chromosomes.
Please note: Wiley is not responsible for the content or functionality of any Supporting Information supplied by the authors. Any queries (other than missing material) should be directed to the *New Phytologist* Central Office.

## Data Availability

Raw sequences have been submitted to NCBI Sequence Read Archive and aggregated under BioProject PRJNA1021599. HapA is NCBI genome GCA_036321655.1 and HapB is NCBI genome GCA_036321645.1. Individual sample accession numbers can be found in the Supporting Information accompanying this article (Tables S1, S2). Novel scripts used in the analyses underlying this article, functional gene annotations for the white oak genome, and additional datasets and output are available from Zenodo doi: 10.5281/zenodo.14736109.
